# He Laid down His Life

**Published:** 1887-02-05

**Authors:** C. Grant-Furley


### "He Laid Down his Life;" a Dispensary Story.

By C. Grant-Furley.

It would be a fine thing to be twenty-one again, if only for the sake of being thoroughly in earnest over anything—work or play, love, hate, jealousy, or ambition.

This reflection, which comes to my mind with increasing frequency as middle age makes me more disposed to take things easily, and yield to the "eternal law of compromise," always brings to my mind the story of Philip Dare.

He was the most thorough man I ever met, intensely energetic in everything he took up. Some people said he was idle; but the fact was that such a good-looking, good-natured fellow as he, was bound to be popular, and he had often so much waltzing, and flirting, and tennis to do that he really hadn't time for study. And after all he had the power, after neglecting his work for six months, of burying himself in his books and getting up enough information in six weeks to take him through an examination with credit—a dangerous facility. I was hardly one of his intimates, though we were studying medicine at the same time, for he was a senior student when I began my course; and I became acquainted with him only through sharing the rooms of John Maclaren, who was his great chum.

I don't know what drew Maclaren and Dare together, unless it was the attraction of opposites. Poor and plain, without an acquaintance in the town, and devoted to his studies to the exclusion of all other interests, Maclaren was the direct antithesis of his friend; but the two hung together in a wonderful way, and thought no end of each other's talents.

"If Dare would only take the trouble to work for three or four hours a day," said Maclaren, "he would carry off every prize and scholarship in the place;" while Philip told me, nearly every time I saw him alone, that "old Jack would be at the top of the profession before he was forty; for he had more brains than any half-dozen ordinary fellows put together."

I won't deny that sometimes Maclaren and I got tired of the dulness of our attic lodging, and would have liked to see something more attractive in the way of womankind than our elderly landlady; and Philip, suspecting this, managed, like the good fellow he was, to get us an invitation to Dr. Bovell's.

Dr. Bovell was one of our examiners, therefore a person whom one wanted to be on good terms with, and he had a delightful wife and a very pretty and charming daughter. Dare's father had been one of Dr. Bovell's college friends, so Philip had the run of the house, and was an immense favourite with the whole family, as indeed, he was with everybody. He was there continually, and somehow, without his saying anything definite on the subject, Maclaren and I came to know that he was in love with Miss Bovell. Therefore, when the invitation came for us to dine there, we went in some curiosity to see if his goddess was at all worthy of our Philip.

Well, she was; it wasn't merely that she was pretty, but she had a way of appearing so interested in all you said and did, that no man possessed of the average amount of vanity could possibly resist her. I think Gladys Bovell had the most winning manners of any woman I ever met.

I fancy it was because he looked so grave and unattractive that she paid so much attention to Maclaren; but perhaps Philip had told her about him and his hard, silent, studious life. Whatever was the reason, she took great pains to make the evening pass agreeably for him.

She had a touch of originality about her that seemed to strike Maclaren, who, I don't think, would have got on very well with a conventional young lady.

"You are not one of those who call my father a brute, I hope, Mr. Maclaren?" she asked, quite calmly. "That is how he was described to me the other day."

"I am surprised anyone should speak of him so," answered John,—"above all, to you."

"Oh! I must do the poor man justice. He didn't know who I was, and he was writhing under the agony of having failed in his examination—a failure due entirely, he said, 'to that brute Bovell.'"

"How did you receive the information?" asked John, with some amusement.

"Sympathetically. I inquired into his woe, and found that his grievance was this: He had failed to answer the first six or seven questions put to him in his oral examination; he admitted that he wasn't very well up in the subject, and at last papa lost patience, and producing a visiting-card, said to him: 'Mr. Brown, will you be kind enough to put down on that card all that you *do* know of the science of medicine?' I could not help laughing, for the remark was just in papa's style, and I could almost fancy I heard him say it; but I think that, on the whole, I behaved well to the miserable man, for I let him leave me without informing him that he was confiding all this to 'the brute's daughter.' Did papa ever ask you to set down all you know on a visiting-card?"

"No; he never gave me credit for so much power of condensation," answered Maclaren, smiling. "The brutality of Dr. Bovell's character has always been hidden from me; and I don't think you could find many men to endorse Mr. Brown's statement."

I can't recollect ever seeing Maclaren smile before this moment, and it seemed to alter his face wonderfully, indeed, to soften and transfigure the whole man. I could not be mistaken in the change, for I saw that Miss Bovell noticed it too. She had been talking in a light, ordinary tone, but when, after looking at him for a moment with a wondering expression on her face, she essayed some commonplace remark, her voice took a lower tone and faltered in the utterance, while her eyes drooped and the colour slightly rose in her cheeks.

Philip, who had been observing delightedly how well his friend and his sweetheart were getting on together, seemed a little vexed that after this they rather avoided each other, but for my part I suspected mischief from that moment.

To state the matter briefly, Maclaren fell in love with Gladys Bovell at first sight. I was sorry for him; for of course I never dreamed of her preferring him to our gay, handsome Philip. And yet that was just what happened. They did not see each other often, and I am sure John never meant to cut out his friend; but it is strange how, when two people love each other, the love will betray itself in eyes and voice, in looks and words and silence, and how some day it will inevitably reach distinct utterance.

One day Maclaren came in and told me very quietly, but with a wonderful light in his eyes: "Something very strange has happened to me. It seems almost miraculous—too good to be true. Gladys Bovell loves me."

I did not think, as perhaps most people would have done, of Maclaren's lack of money and influence, nor ask myself if Dr. Bovell would not have preferred some other for his son-in-law; I knew my friend, and that knowledge was answer sufficient for all doubts. But I did think and say—"What about Dare?"

"That's the hard bit," he answered; "the one flaw in my happiness, to think I have been preferred to Dare. But I could not help it, and even if Gladys had not cared for me, she would not have taken him." And though he did not say say so, I understood that Philip had already asked Gladys to marry him and been refused.

I don't know how the news came to Philip's ears, but the next evening he burst in on us in hot wrath, as impulsive in his anger as he used to be in his happiness, but so wild with passion as to be almost beyond recognition.

He called Maclaren every evil name he could think of—coward, sneak, false friend, mercenary self-seeker. John bore it all in silence; one would almost have thought he deserved the titles. But when Dare began to call Gladys a coquette, he spoke at once.

"Be quiet!" he cried. "I'll take a good deal from you, Dare, and I know this is all bitterly hard on you, but not even you shall say a word against her."

Philip said nothing more, but he made a rush at Maclaren's throat. The latter—I didn't think he had so much strength in him—just caught hold of him in his arms, and dragged him out of the room. He almost flung him downstairs, and as he closed and locked the door, he said, as quietly as if it were an ordinary greeting, "You don't come here again while you have a disrespectful word to say of Gladys Bovell."

"I hope I'll see you dead before you marry her!" cried Philip bitterly; and then there was a thundery silence.

About an hour after Dare had left us, a knock came to the door. It was a messenger asking for him. One of his patients had sent for him. Both he and Maclaren were doing dispensary work at the time, and were always being called out to see some miserable creature, in some of the filthiest dens in the town; and as he was not at home, his landlady had sent to ask if he were at our rooms, where hitherto, indeed, he had usually been to be found. The reply, of course, was that Mr. Dare was not with us, and that we did not know where he could be found; but Maclaren made a few inquiries as to the nature of the case he was wanted for.

"I think I'll go myself," he said finally.

"I don't see why you should," said I. "It's Dare's work, and not yours; it appears to be an infectious business; and I don't suppose you are particularly set on bearing his burdens for him just at present."

For, you see, though I liked Dare, and was proud of his friendship, I had a special worship of Maclaren. I thought the other was doing him an injustice.

"I don't know that I am justified in letting a fellow-creature die just because I have a spite against the man who ought to be attending him," answered John in a quiet way, as if he was thinking out the question in his own mind; and in spite of my remonstrances off he went.

He was absent for several hours. "It was as bad a case of diphtheria as I ever saw," he told me when he came back. "I performed tracheotomy—the first time I ever did it entirely myself—and I think things will go all right now. I must have been nervous too—perhaps I'm not quite so cool as usual to day—for I gave myself a nasty scratch with the lancet just as I finished the operation."

A very nasty scratch it proved to be; for in a few days Maclaren was laid up with blood-poisoning, and finally diphtheria declared itself. It was a bad case, about as bad as I ever saw; and Dr. Bovell, who attended him, looked very grave over the matter.

I was terribly anxious. So, you may guess, was some one else, who daily sent flowers and loving messages by her father. It was fine to hear Dr. Bovell, who did not altogether approve of the match—thought his daughter might have done better—conscientiously repeating the tender phrases to the sick man, and trying every possible means to pull him through.

But all our anxiety was nothing to Philip Dare's. He looked ghastly, as if he were dying himself; but he was Maclaren's most skilful and untiring nurse, far surpassing the professional one who had been sent.

In vain I begged him to take some rest. "I can't rest," he said. "Don't you understand that I feel like Cain? I wished for his death; and now he's dying, I feel like a murderer."

I argued against this morbid nonsense; but I couldn't argue it out of his head. He simply wore himself out with remorse. He was so awfully in earnest about everything he did.

At last there came a day when Gladys was brought to see her lover, because it was likely that she might never again see him in life. Maclaren was unconscious. He did not know with what an agonised look the fair face bent over him, nor guess how she desired to take death from his lips in a kiss; but Dare, who was there, saw it all.

Dr. Bovell took his daughter away. As she passed Philip she caught his hand and said, "Save him for me."

He bowed. "If I can I will," he answered, quite calmly, as if it were an every-day occurrence to drag a man back from the jaws of death. The doctor put Gladys in his carriage and sent her home; then came back to the sick-room.

"We must put in a tube," he said, quickly. "It is his only chance." So the operation which Maclaren had been so pleased to have succeeded in a short time before, was now performed on himself.

It did not promise to be successful this time. The coat of mucus in the throat was so thick that the air could not reach the wind-pipe, and it seemed as if he must die.

"I feared this," said the doctor.

"It isn't hopeless yet," remarked Dare. "I will suck the stuff through the tube and clear the throat."

"My dear boy, do you know what you are suggesting? It's a most risky thing, almost certain death. I should feel answerable to your father if anything happened to you."

"I can't let Maclaren die—for the sake of Gladys," answered Philip; and he almost pushed the doctor aside, and knelt by Maclaren's bed.

In a few moments the thing was done, and almost immediately we could perceive the relief the patient experienced, and very soon he began to regain strength. Dare had saved his life.

But at the cost of his own! He caught the disease, and, somehow, before we had even begun to regard it as a serious case, he was dead. He had not much stamina to back that bright, joyous, energetic temperament of his; and he collapsed at the first onset of the disease.

Well, that's all. Gladys married Maclaren in due time, and they were happy and successful. They did not speak much of Philip Dare; but they always kept a tender and reverent recollection of him in their minds; and it was Gladys who desired that on his tombstone should be engraved the words I have taken for the title of this short history: "He laid down his life." I think they are appropriate.

